# Design of the muscles in motion study: a randomized controlled trial to evaluate the efficacy and feasibility of an individually tailored home-based exercise training program for children and adolescents with juvenile dermatomyositis

**DOI:** 10.1186/1471-2474-13-108

**Published:** 2012-06-21

**Authors:** Esther A Habers, Marco van Brussel, Anneli C Langbroek-Amersfoort, Annet van Royen-Kerkhof, Tim Takken

**Affiliations:** 1 Child Development & Exercise Center, Wilhelmina Children’s Hospital, University Medical Center Utrecht, Room KB.02.056.0, P.O. Box 85090, NL-3508 AB, Utrecht, the Netherlands; 2 Faculty Center for Human Movement Sciences, University of Groningen, University Medical Center Groningen, Groningen, the Netherlands; 3 Department of Pediatric Immunology and Rheumatology, Wilhelmina Children’s Hospital, University Medical Center Utrecht, Utrecht, the Netherlands

**Keywords:** Exercise, Children, Randomized controlled trial, Juvenile dermatomyositis

## Abstract

**Background:**

Juvenile dermatomyositis (JDM) is a rare, often chronic, systemic autoimmune disease of childhood, characterized by inflammation of the microvasculature of the skeletal muscle and skin. Prominent clinical features include significant exercise intolerance, muscle weakness, and fatigue. Despite pharmacological improvements, these clinical features continue to affect patients with JDM, even when the disease is in remission. Exercise training is increasingly utilized as a non-pharmacological intervention in the clinical management of (adult) patients with chronic inflammatory conditions; however no randomized controlled trials (RCT) have been performed in JDM. In the current study, the efficacy and feasibility of an exercise training program in patients with JDM will be examined.

**Methods/design:**

Subjects (*n* = 30) will include 8–18 year olds diagnosed with JDM. The intervention consists of an individually tailored 12-weeks home-based exercise training program in which interval training on a treadmill is alternated with strength training during each session. The program is based on previous literature and designed with a defined frequency, intensity, time, and type of exercise (FITT principles). Primary outcome measures include aerobic exercise capacity, isometric muscle strength, and perception of fatigue. The study methodology has been conceived according to the standards of the CONSORT guidelines. The current study will be a multi-center (4 Dutch University Medical Centers) RCT, with the control group also entering the training arm directly after completion of the initial protocol. Randomization is stratified according to age and gender.

**Discussion:**

The current study will provide evidence on the efficacy and feasibility of an individually tailored 12-week home-based exercise training program in youth with JDM.

**Trial registration:**

Medical Ethics Committee of the University Medical Center Utrecht, the Netherlands: 11–336; Netherlands Trial Register (NTR): NTR 3184.

## Background

The pediatric forms of inflammatory idiopathic myopathies (IIM) embody a group of rare systemic autoimmune conditions, characterized by chronic skeletal muscle inflammation with an age of onset of <18 years. Despite being a rare condition, juvenile dermatomyositis (JDM) is the most common type of pediatric IIM, representing about 85% of IIM diagnoses in childhood [1]. JDM is characterized by inflammation of the microvasculature of the skeletal muscle and skin. While the etiology of the disease remains largely unknown, environmental and genetic factors are thought to play a role in its development [2]. Prominent clinical features are significant muscle weakness [3], anaerobic- and aerobic exercise intolerance [4-8], and fatigue [9]. Furthermore, the patients show a skin rash commonly seen on the face and extremities [10]. Less frequently, the gastrointestinal tract, lungs, heart, and rarely the central nervous system may also be involved [10-12]. Despite pharmacological improvements, these clinical features are persistently experienced in patients with JDM, even when the disease is in remission [12-14].

Exercise training is increasingly utilized as a non-pharmacological intervention in the clinical management of patients with chronic inflammatory conditions. As opposed to typically developing healthy children, those with chronic illness are often restricted in their participation in physical activity as a consequence of real or perceived limitations imposed by their condition. The chronic condition itself often causes hypoactivity leading to a deconditioning effect and a reduction in functional ability, which ultimately results in a downward spiral of further hypoactivity and deteriorating health [15]. Further, children who are hypoactive are also thought to be at high risk of developing a number of secondary health complications commonly associated with a sedentary lifestyle including cardiovascular disease, obesity, and pre-diabetes [15]. Exercise training might prevent or slow down the deconditioning as a result of hypoactivity, but it might also reduce the systemic inflammation. To date, very little is known about the effects of exercise training in chronic pediatric conditions, including JDM. In fact, only one study has examined the effect of exercise training in one child with JDM in remission. Omori et al. reported improvements in muscle strength, aerobic exercise capacity, and muscle function in this child after completing a 16-week exercise training program [16]. In adults with active as well as inactive stable dermatomyositis, exercise training has been shown to improve aerobic exercise capacity, muscle strength, functional ability, fatigue, and health status [17]. These improvements may theoretically be explained by the role of chronic exercise in inducing changes at the origins of the observed exercise intolerance, which include decreased capillary density, perivascular atrophy of type I and II muscle fibers, and reduction of key enzymes essential for producing ATP [18].

Studies in patients with adult and juvenile dermatomyositis suggest that exercise training is safe since it has not been associated with any increases in the degree of inflammation after a single bout of exercise in JDM [19], or after an exercise training program in one patient with JDM [16], as well as in a number of adult patients with dermatomyositis [17].

While there is limited evidence to support the role of exercise training in the management of patients with JDM, definitive evidence is still lacking. In fact, to our knowledge, no randomized controlled trials (RCT) of exercise training in JDM have been performed, which may be secondary to limited patient availability and other feasibility issues.

As such, we designed a RCT to study the feasibility of an individually tailored 12-week home-based exercise training program, and to assess its efficacy in children and adolescents with JDM. The wash-out effects of this program after another 12 weeks will also be examined.

## Methods/design

### Study design

**Figure 1:**
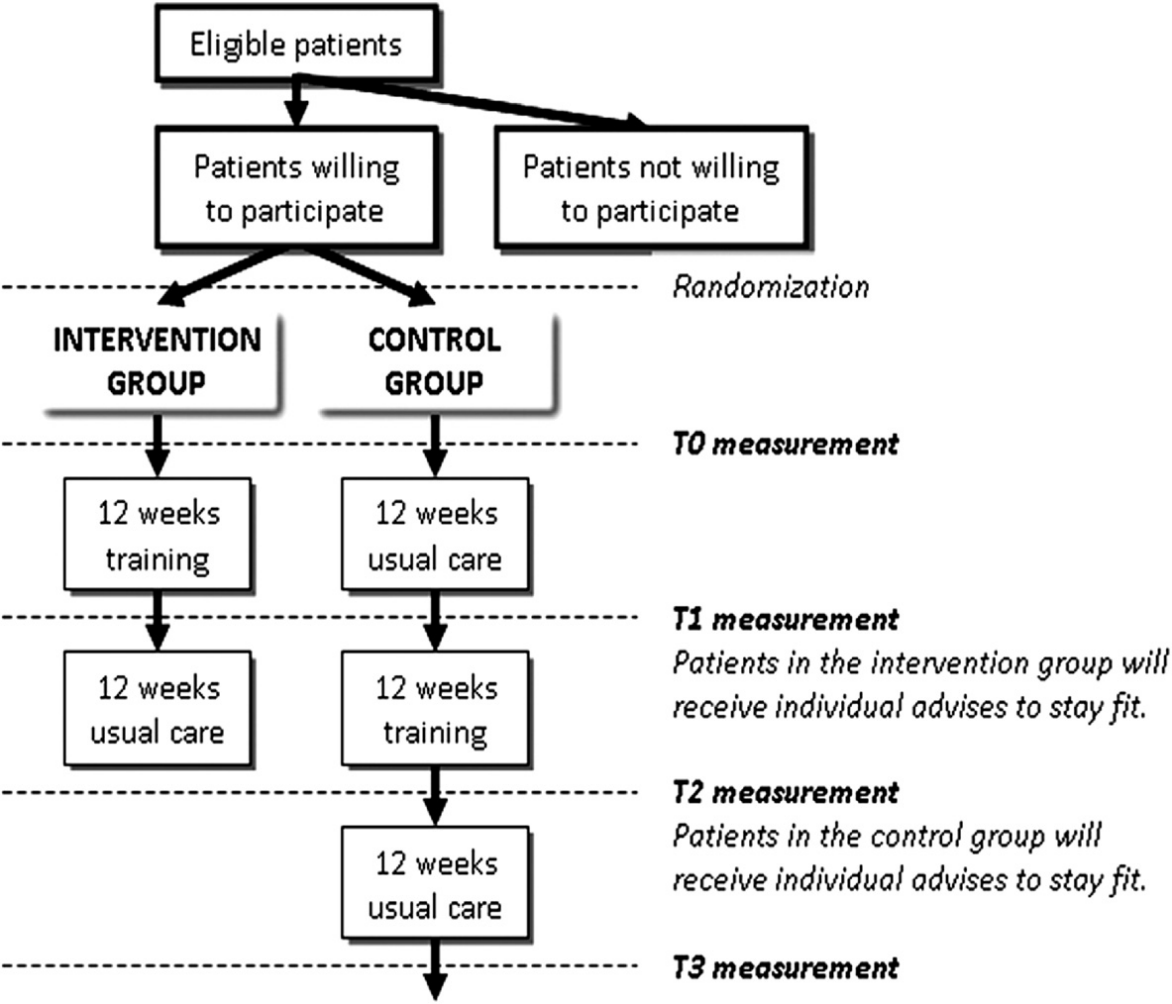
Flow diagram of the study design.

The current study is a multi-center (4 sites) randomized controlled trial, with the control group also entering the training arm directly following completion of the initial protocol. After randomization (1:1), subjects in both groups will perform a test battery of exercise tests, questionnaires, and other measurements (see study outcomes) (T0 measurement). Thereafter, subjects in the intervention group will receive an exercise training regimen, while the subjects in the control group will continue to receive standard care over a 12-week period. Subsequently, subjects in both groups will repeat the battery of tests from T0 (T1 measurement). Subjects in the intervention group will then receive individual advice based on their T1 results and educated on the physical activity guidelines for children [20] in order to help maintain fitness levels following the intervention. Subjects in the control group will enroll in the exercise training program. Following this 12-week period, subjects in both groups will repeat the battery of tests from T0 and T1 (T2 measurement) and the subjects in the control group will receive individual feedback in the same manner as the intervention group at T1. After 12 weeks, the subjects in the control group will repeat the battery of tests from T0, T1, and T2 (T3 measurement). See Figure1 for a flow diagram of the study design.

### Subjects

All patients diagnosed with JDM by a pediatric rheumatologist/immunologist according to the Bohan and Peter criteria between the ages of 8 and 18 years from 4 outpatient clinics in the Netherlands will be assessed for eligibility. The 4 outpatient clinics include the Wilhelmina Children’s Hospital University Medical Center (UMC) in Utrecht, the Beatrix Children’s Hospital UMC in Groningen, the Sophia Children’s Hospital Erasmus Medical Center in Rotterdam, and the St. Radboud Children’s Hospital UMC in Nijmegen.

Patients will be excluded for eligibility upon (1) a medical status that contraindicates any form of exercise testing; (2) an insufficient understanding of the Dutch language in the patient and/or the parents/caregivers; (3) the presence of medical events that might intervene with the outcome of testing and/or the trial (such as a planned operation) and/or (4) a negative advise from the rheumatologist and/or physiotherapist based on resent relapse or no limitations in exercise tolerance (in other words: the patient was either not good enough or too good). All eligible patients will be asked to participate in the study. All parents/caregivers as well as subjects >12 years of age years must provide informed consent before enrolment in this study, which is approved by the Medical Ethics Committee of the UMC Utrecht, the Netherlands.

The flow of the subjects in the study will be depicted in the CONSORT Flowchart format [21]. The reasons for non-eligibility, declination to participate, and non-completion of the study will be reported in this flowchart as well.

### Exercise training program

The intervention comprises of an individually tailored 12-week home-based exercise training program, combining interval training on the treadmill (DKN Technology, T210, Belgium) with strength training. Both types of training are selected based on recommendations for patients with chronic conditions [22], as well as to specifically target the impaired exercise capacity and muscle strength observed in patients with JDM. The home-based nature of the intervention is selected to minimize the burden and costs associated with travel for both the patients and their parents. The training program is divided in 3 phases of 4 weeks each as shown in Table1. Concerning the FITT factors (frequency, intensity, time, and type) [23] and additional parameters, the following selections are made.

**Table 1:** Description of the exercise training program

**Phase**	**1**	**2**	**3**
*Aim*	- Familiarizing the patient with the treadmill	- Improving aerobic exercise capacity	- Improving aerobic exercise as in phase 2 albeit with a higher intensity
	- Improving aerobic exercise capacity	- Improving muscle strength	- Improving muscle strength as in phase 2
	- Developing proper strength training techniques		
*Frequency [week*^*-1*^*]*	3	3	2
*Time/session [minutes]*	40-60	45-60	50-60
*Type*	Light interval training on treadmill and strength training.	Moderate interval training on treadmill and strength training.	Vigorous interval training on treadmill and strength training.
**Interval training on treadmill**			
*Intensity [HR as % of* HR_peak_*]*	65-70	70-80	80-90
*Interval duration [minutes]*	3	2-2.5	1-2
*Number of intervals*	4-7	6-10	10-12
**Strength training**			
*Number of exercises*	3	3	3
*Number of sets/exercise*	3	3	3
*Number of repetitions or time/set*	Week 1: 3 repetitions	As much repetitions in 20 or 30 seconds.	As much repetitions in 20 or 30 seconds.
	Week 2–12: as much repetitions in 20 or 30 seconds.		

### Type – interval training on treadmill

The aim of the interval training is to improve the aerobic exercise capacity as it has previously been shown to increase peak oxygen uptake in healthy children and adolescents [24]. Moreover, compared with prolonged continuous training, the short intermittent nature of the exercise better simulates natural physical activities of children [25] and it is more likely to be perceived as enjoyable in a pediatric population. During the progression of the exercise training program, the intensity of the intervals will increase simultaneously with a decrease in the duration of each bout. The interval training will be performed on the treadmill since walking and running correspond closely to activities of daily living that may be impaired in patients with JDM.

### Type - strength training

The aim of the strength training program is to improve the muscle strength of proximal muscle groups, since these tend to be most affected in JDM. Along with the recommendation that strength training in youth should include large muscle groups [3], the following 3 exercises are included: squat, abdominal crunch, and push-up. To prevent muscle damage, the exercises are concentric in nature and against gravity (no additional weight lifting). A ball is implemented in some of the exercises to maintain the playfulness of the exercise.

Interval training on the treadmill and strength training will be performed in an alternating fashion during each session.

### Frequency

In order to improve aerobic exercise capacity and muscle strength in children and adolescents, a training frequency of 3-4 [24] and 2-3 [24,26] times per week, respectively, is recommended. By taking these guidelines into account, as well as the time requirement for the patient and parents to complete the session, the training frequency during phase 1 and 2 is set at 3 sessions per week and it is reduced to 2 times per week during phase 3 together with an increase in both intensity and session duration. Consequently, the total training program consists of 32 training sessions. Subjects will be instructed to perform these sessions on non-consecutive days.

### Intensity – interval training on treadmill

It is important that the intensity of the exercise training is similar in all subjects [24]. Because of the large inter-individual variability in peak heart rate (HR_peak_) among children [24], the intensity of the treadmill training in the current study will be defined by HR as percentage of HR_peak_, which will be assessed prior to training at T0 (intervention group) or T1 (control group). In this way, the standardization of the intervention is guaranteed.

Baquet et al. [24] state that training at a HR > 80% of HR_peak_ may be essential in order to improve aerobic exercise capacity in healthy children and adolescents. However, this threshold intensity is likely to be reduced for most deconditioned patiens with a chronic condition. Durstine et al. [22] suggested a training intensity with an oxygen uptake of 10-60% of peak oxygen uptake in these chronic patients, which corresponds to approximately a HR of about 66% of HR_peak_[27]. Taking these guidelines into account, exercise intensity in the current training program will begin at a HR of 65% of HR_peak_ and increase to a HR of 90% of HR_peak_ by the end of the program. Further, periods of high intensity exercise (3–1 minutes at HR of 65-90% of HR_peak_) will alternate with short periods of low intensity exercise (1 minute at a HR of 50-60% of HR_peak_).

The intensity of the intervals is ascertained in advance (see Table 1) and it is constant for intervals during a single training session. The initial gradient of the treadmill during the intervals is set at 0% for each subject. The speed will be adjusted individually in such a way that the HR during the intervals is approximately 65% of HR_peak_. This combination of gradient and speed is called level 1. In total, 24 levels are formulated in which the next level is either characterized by an increase in speed (alternately 0.6 and 0.4 km·h^-1^) or an increase in gradient of 1%.

During the intervals, subjects and parents will monitor their HR using a heart rate monitor (DKN Technology, Belgium). If HR does not fall within the predefined training range, the intensity of the next training session will be increased or decreased by 1 level so as to maintain the proper intensity.

### Intensity - strength training

For strength training in healthy children and adolescents, 1–3 sets of 6–15 repetitions are recommended [26,28]. In the current training program, each of the 3 exercises consists of 3 sets. During the first week, only 3 repetitions are prescribed per set so as to ensure the subjects develops proper strength exercise techniques [26]. Over the course of the subsequent 11 weeks, the intensity of the strength training is set by time. Squats will be performed for 30 s per set and both the abdominal crunches and push-ups will be performed for 20 s per set. All exercises have 2 possible extensions in which the exercise load becomes heavier and more challenging. An extension will be prescribed when the subject shows proper techniques and describes the exercise as light or easy.

Subjects will be given a 1 min rest period between sets according to the recommendations of Faigenbaum et al.[26]. Such a short period allows for the development of muscular endurance [29], which is highly desired in this patient group.

### Time and duration

The total time per session, which includes treadmill training, strength training, warming-up, cooling-down, and rest periods, is 40–60 minutes. Each session consists of 15–25 minutes at or above the threshold intensity, as recommended by Takken et al. [23] The program duration is set at 12 weeks according to the recommendations for exercise training in children with chronic conditions [30].

### Warming-up and cooling-down

Each exercise session starts and ends with a 5-min during low intensity walk on the treadmill at a HR of 50-60% of HR_peak_[26].

### Supervision

Each subject will receive a detailed and individualized description of the exercise program and will be asked to keep track of every completed stage. Parents will be asked to be present at each training session to provide their child with support and motivation, to ensure proper strength training techniques, as well as to assist them with the use of the treadmill and the HR monitor. Furthermore, the subjects will be asked to keep a day-to-day journal for training compliance. Every 2 weeks, a researcher or physiotherapist will conduct a home visit to observe the training session, to ensure compliance, to motivate the subject, as well as to make adjustments to the training prescription as needed.

### Randomization and blinding

Included subjects will be stratified according to age (≤ or >12 years) and gender after which they will be randomized to either the intervention or the control group. The randomization process will be performed by a person with no clinical involvement in the trial. Blocking is used to ensure close balance of the numbers in each group at any time during the trial. Randomly varying the block size (2 or 4) reserves the unpredictability of the sequence. The sequences will be concealed until interventions are assigned. The researchers performing the assessments, as well as those charged with data analyzes will be blinded to treatment allocation.

### Baseline description of groups

The baseline descriptors of the groups are age, gender, anthropometrics and disease characteristics. The anthropometrics include maturity offset (years), height (M), body mass (kg), body mass index (kg·m^-2^), and fat free mass (kg). The disease characteristics include age at diagnosis (years), disease duration at inclusion (years), disease course (monocyclic/ polycyclic/ chronic), and medication usage. These descriptors will be compared between the treatment group and the control group at T0. Maturity offset will be determined with gender-specific equations including chronological age, height, body mass, and sitting height [31]. Fat free mass will be measured with BODYSTAT® QuadScan 4000. Disease duration at inclusion will be defined as the time from diagnosis to inclusion. Disease course will be classified as monocyclic (no signs of disease activity 2 years after diagnosis), polycyclic (recurrence of active disease after a definite remission (>6 months), followed by one or more flares), or chronic (persistent disease activity >2 years after diagnosis)[32].

### Study outcomes

#### Efficacy

The primary and secondary efficacy outcome measures along with the tools for these assessments are described below and in Table2.

**Table 2:** The primary and secondary study outcomes and the accompanying measurement tools used

	**Items**	**Range**	**+/−**
**Aerobic exercise capacity***(Maximal exercise test on treadmill)*			
VO_2peak_ [l·min^-1^]	na	>0	+
VO_2peak/kg_ [ml·kg^-1^·min^-1^] ^1^	na	>0	+
O_2_ pulse [ml·beat^-1^]	na	>0	+
Endurance time [min]	na	>0	+
VO_2VAT_ [l·min^-1^]	na	>0	+
VO_2VAT/kg_ [ml·kg^-1^·min^-1^]	na	>0	+
**Isometric muscle strength***(Hand-held dynamometry – Break method)*			
Maximal force right knee extensors [N]	na	>0	+
Maximal force left knee extensors [N]	na	>0	+
Maximal force right hip flexors [N]	na	>0	+
Maximal force left hip flexors [N]	na	>0	+
**Perception of fatigue***(PedsQL Multidimensional Fatigue Scale – patient form)*			
Total score	18	0-100	+
Subscale *General Fatigue* score	6	0-100	+
Subscale *Sleep/Rest Fatigue* score	6	0-100	+
Subscale *Cognitive Fatigue* score	6	0-100	+
**Muscle soreness***(10-cm Visual Analogue Scale muscle soreness)*			
Visual Analogue Scale muscle soreness [cm]	na	0-10	-
**Muscle function***(BOT-2 Subscale 8 – Strength)*			
Total score	5	0-42	+
Item *Distance standing long jump* [m]	na	>0	+
Item *Amount of sit-ups in 30 s*	na	≥0	+
Item *Amount of push-ups in 30 s*	na	≥0	+
Item *Time wall sit* [s]	na	0-60	+
Item *Time V-up* [s]	na	0-60	+
**Muscle function***(Childhood Myositis Assessment Scale)*			
Total score	14	0-52	+
**Functional sub maximal aerobic exercise capacity***(6-Minute Walk Test)*			
Distance [m]	na	>0	+
**Physical activity enjoyment***(Physical Activity Enjoyment Scale)*			
Total score	17	17-119	+
**Quality of life***(PedsQL Generic Core Scale – patient form)*			
Total score	23	0-100	+
Subscale *Physical Functioning* score	8	0-100	+
Subscale *Emotional Functioning* score	5	0-100	+
Subscale *Social Functional* score	5	0-100	+
Subscale *School Functioning* score	5	0-100	+
**Functional ability***(Childhood Health Assessment Questionnaire 30 + 8)*			
Subscale *Dressing* score	4	0-3	-
Subscale *Arising* score	2	0-3	-
Subscale *Eating* score	3	0-3	-
Subscale *Walking* score	2	0-3	-
Subscale *Hygiene* score	5	0-3	-
Subscale *Reach* score	4	0-3	-
Subscale *Grip* score	5	0-3	-
Subscale *Activities* score	5	0-3	-
Subscale *School/Extracurricular* score	8	0-3	-
Disability score [%]	38	0-100	-
Visual Analogue Scale pain [cm]	na	0-10	-
Visual Analogue Scale global disease severity [cm]	na	0-10	-
**Physical activity***(Actical accelerometer for 7 days)*			
	na	>0	+
**Physical activity***(Activity journal for 2 weekdays + 1 Saturday)*			
Time inactive [%]	na	0-100	-
Time light active [%]	na	0-100	−/+
Time moderate active [%]	na	0-100	−/+
Time vigorous active [%]	na	0-100	+

Abbreviation: na: not applicable.

^1^ the primary outcome measure on which power calculation was performed.

+: an increase in value denotes an improvement -: a decrease in value denotes an improvement.

### Primary study outcomes

*Aerobic exercise capacity* will be measured with a treadmill-based (RAM, Accuramed BVBA, Lummen, Belgium) incremental maximal exercise test according the protocol set out by the German Society for Pediatric Cardiology [33]. Briefly, the test starts at a speed of 2 km·h^-1^ with a 0% grade; the speed increases by 0.5 km·h^-1^ and the grade by 3%, up to a maximum of 21%, every 90 s. The test is terminated upon voluntary exhaustion, despite strong verbal encouragement.

Throughout the test, the subject will wear a facemask (Hans Rudolph Inc, Kansas City, MO) connected to a gas analysis system (ZAN 600, Accuramed BVBA, Lummen, Belgium), which will allow for a sample of expired gases to pass to a flowmeter, an oxygen analyzer, and a carbon dioxide analyzer. Volume calibration and gas analysis calibration will be performed before each testing session. The flow meter and gas analyzers are connected to a computer that calculate breath-by-breath minute ventilation (VE), oxygen uptake (VO_2_), carbon dioxide output (VCO_2_), and the respiratory exchange ratio (RER = VCO_2_/VO_2_) using conventional equations. HR will be measured continuously via a 12-lead electrocardiogram (CardioPerfect, IT medical, the Netherlands). The test will be deemed maximal when the HR is >180 beats·min^-1^ and/or the RER >1.0.

The outcome measures related to aerobic exercise capacity that will be assessed include: VO_2peak_ [l·min^-1^]: the average volume of the absolute oxygen uptake during the last 30-s period of the test; VO_2peak/kg_ [ml·kg^-1^·min^-1^]: the absolute VO_2peak_ divided by body mass. This variable was used for the power calculation; VO_2VAT_ [l·min^-1^]: the absolute VO_2_ eliciting the ventilatory anaerobic threshold (VAT). The VAT will be determined as defined by an increase in both the ventilatory equivalent of oxygen (=VE/VO_2_) and end-tidal pressure of oxygen with no concurrent increase in the ventilatory equivalent of carbon dioxide (=VE/VCO_2_); VO_2VAT/kg_ [ml·kg^-1^·min^-1^]: the absolute VO_2VAT_ divided by body mass; O_2_ pulse [ml·beat^-1^]; and endurance time [min]: the time from the start to the end of the protocol.

*Isometric muscle strength* of the proximal muscle groups in the lower extremities will be measured on both the right and left sides with a calibrated CITEC hand-held dynamometer (C.I.T. Technics, Groningen, the Netherlands). Both the extensors of the knee joint and the flexors of the hip joint will be assessed with 3 consecutive measurements according to the ‘break method’, wherein the examiner gradually overcomes the muscle force and stops at the moment the extremity gives way [34]. The highest value of the 3 repetitions will be recorded. All within-subject tests will be performed by the same assessor using the same hand-held dynamometer to prevent inter-observer and inter-instrument bias [34].

*Perception of fatigue* will be measured with the Dutch translated PedsQL Multidimensional Fatigue Scale. This scale, designed to measure fatigue in pediatric patients, reflects 3 subscales: general fatigue, sleep/rest fatigue, and cognitive fatigue [35-37].

### Secondary outcome measures

*Muscle soreness* will be measured using a 10-cm Visual Analogue Scale (VAS). *Muscle function* will be assessed via Subscale 8 (Strength) of the Bruininks-Osteretsky Test of Motor Proficiency, Second Edition (BOT-2), which include 5 items that are related to the strength training program of the subjects [38]. The Childhood Myositis Assessment Scale (CMAS) is specially designed to assess the functional consequences of proximal muscle strength and endurance, and will also be utilized as a marker of *muscle function* in our subjects [39]. *Functional sub maximal aerobic exercise capacity* will be assessed by examining walking distance at the 6-min walk test (6MWT) [40]. This will be followed by an assessment of *physical activity enjoyment* using the Physical Activity Enjoyment Scale (PACES) [41]. *Quality of life* will be measured with the patient form of the Dutch translated PedsQL Generic Core Scale [36,42,43]. *Functional ability* will be measured with the Childhood Health Assessment Questionnaire (CHAQ), a disease specific questionnaire that measures the grade of disability in performing activities of daily living as observed by the parents [44-46]. The VAS discomfort items of the CHAQ will be filled in by the subjects. Finally, habitual levels of *physical activity* will be monitored via accelerometry (Actical) [47] and a Bouchard activity journal [48,49] over a 7 day period.

### Power calculation

A power calculation was performed based on previously published data relating to one of the primary outcome measures: VO_2peak/kg_. The mean ± standard deviation VO_2peak/kg_ of patients with JDM (in remission) is 33 ± 8 ml·kg^-1^·min^-1^[13]. The aim of this training intervention is to improve VO_2peak/kg_ by at least 22%, which is the weighted mean improvement in exercise training studies in adults with inflammatory myositis [17]. Given an 80% probability of detecting a treatment effect, with significance set at 0.05 (two-tailed), the required number of total subjects is 22; 11 in each of the 2 groups. Upon factoring in a 20% (*n* = 5) chance of missing observations and/or drop-outs, the total sample size increase to 27 patients. For simplicity in the randomization process, we want to include 30 patients.

### Statistical analysis

Data will be checked for normal distribution. Three different types of analysis will be performed.

#### Intervention group Vs control group

To examine the efficacy of the intervention, the outcome measures of the intervention group and control group will be compared at each of time points T0 and T1 using mixed linear regression analysis with group (intervention/control) as independent variable and the outcome measures (see Table2) as separate dependent variables.

#### Intervention in control group

To examine the efficacy of the intervention in the control group, changes in outcome measures between T0 and T1 (usual care) will be compared with the changes in outcome measures between T1 and T2 (intervention) using a paired samples *T*-test.

#### “Wash-out” effect in intervention and control group

Repeated measures ANOVA analyzes will be performed to examine whether the effects of the intervention are maintained after 12 weeks. Specifically, the outcome measures of both groups will be compared between post-12-week usual care after intervention vs. post-intervention and post-12-week usual care after intervention vs. pre-intervention.

Results will be presented as linear regression coefficients representing mean group differences with their corresponding 95% confidence intervals (CI). Statistical significance will be defined as cases in which the 95% CI does not include the null value. Nominal variables will be analyzed using chi-square analysis. All results will be analyzed using the modified intention to treat approach which means that all subjects will be included in the groups to which they are randomized, and the researchers make efforts to obtain outcome data for all subjects, even if they do not complete the full intervention [50].

### Feasibility

Feasibility will be assessed by examining the amount of training sessions each subject performed during the intervention. Every week, the subject has to start the exercise training program of a new week, independent whether the proposed amount of training sessions of the previous week is reached. When the subject is not able to train for at least 7 consecutive days (*e.g.* due to illness or holiday), the subject will be asked to resume the exercise training program where he/she was ended.

## Discussion

This paper presents the design of a randomized controlled study that will provide evidence on the efficacy and feasibility of an individually tailored 12-week home-based exercise training program for children and adolescents with JDM. The wash-out effects of this program after another 12 weeks will also be examined.

Among the many strength of this study is first, the fact that this will be the first RCT to assess the effects of exercise training in patients with JDM. Second, evidence from literature is used to design the treadmill training as well as the strength training program. Third, the focus of the intervention, as well as the primary outcome measures, corresponds to the major clinical concerns of patients with JDM. Finally, and perhaps most importantly, the intervention is a home-based program, which minimizes the load for the patients and their parents while likely stimulating the patient’s desire and motivation to continue exercising upon completion of the intervention.

The study methodology has been conceived according to the standards of the CONSORT guidelines [21].

## Competing interests

The authors declare that they have no competing interests.

## Authors’ contributions

All authors contributed in designing, drafting, and revising the manuscript, and they all have given approval of the final version. All authors read and approved the final manuscript.

## Pre-publication history

The pre-publication history for this paper can be accessed here:

http://www.biomedcentral.com/1471-2474/13/108/prepub
